# Supplementary material for: Are carbon emissions trading and green financial instruments synergistic? -Comprehensive quantitative research based on content analysis
Source: PLoS One. 2024 Mar 7;19(3):e0298601. doi: 10.1371/journal.pone.0298601 (PMC10919601; doi:10.1371/journal.pone.0298601)
Supplement: S1 Data — (ZIP) [file pone.0298601.s001.zip › Data/地级市Z_F工作报告.zip!/░▓╗╒/═¡┴Ω╩╨/└┤╫╘╣1⁄2╓┌║┼í╛┬φ┐╦╩2╛▌═°í┐_2012.docx]

各位代表：
  现在，我代表市人民政府，向大会报告工作，请予审议，并请市政协委员和其他列席人员提出意见和建议。

一、2011年工作回顾

    刚刚过去的2011年，全市人民在省委、省政府和中共铜陵市委的坚强领导下，认真贯彻党的十七大和十七届三中、四中、五中、六中全会以及省、市第九次党代会精神，牢固树立科学发展观，积极应对复杂严峻的宏观环境，扎实推进保增长、调结构、惠民生、促和谐等各项工作，转型跨越迈出新步伐，幸福铜陵建设取得新成效，实现了“十二五”良好开局。预计，全年地区生产总值580亿元，增长13%。财政收入115亿元，增长30%。社会消费品零售总额117亿元，增长18%。全社会固定资产投资410亿元，增长30%。新增就业2.2万人，城镇登记失业率3.6%。城镇居民人均可支配收入21750元，增长17.5%；农民人均纯收入8700元，增长20%。人口自然增长率3.16‰。节能减排完成省政府下达的各项指标。基本实现了市十四届人大五次会议通过的“54128”年度目标。与此同时，改革取得新突破，继续走在全省、全国前列。城乡一体化综合配套改革率先推进。社区综合管理体制改革得到国务院有关部门充分肯定和社会广泛赞同。创建工作取得重要进展。成为全省第二个国家卫生城市、全国文明城市提名城市、全国残疾人工作示范市、全省唯一的全国科技进步先进市和先进县（区）、全国双拥模范城“三连冠”。这些都为提前八年全面建成小康社会、加快建设幸福铜陵积累了更加坚实的基础。
     一年来，我们主要做了以下工作：
    （一）全力扩大有效投入，经济发展势头稳中趋好。我们坚持把投资作为推动发展的第一抓手。通过依法加强土地征收、优先建设安置房、积极清理闲置土地、推行项目并联审批、完善现场协调督查机制，努力破解项目“落地难”等问题。全年在建项目超过1100个，其中亿元以上项目256个。新开工亿元以上项目58个。皖能铜陵电厂100万千瓦机组、海亮一期等重点项目建成投产。针对经济运行中要素短缺等问题，完善“百名干部进百企”服务机制，及时出台一系列帮扶政策，全年减免企业税费和支持企业发展资金15.5亿元，为中小企业提供担保总额增长38.2%。房地产投资高速增长。完成进出口总额41亿美元，增长20%。经济增长速度逐季攀升，工业企业经济效益综合指数再创历史新高。在全省率先建立物价防控体系，推行地产蔬菜直销和“农超对接”，适时增加粮油储备，新增标准化蔬菜基地3000亩，居民消费价格指数涨幅低于全省平均水平，向困难群众发放物价补贴2000万元，为全省最高。
    （二）加快培育战略性新兴产业，产业转型升级初见成效。我们结合铜陵实际，确定铜基新材料、先进装备制造、节能环保、新能源、现代物流和文化创意为战略性新兴产业，设立引导资金1.5亿元，采取“一个产业、一个规划、一批项目、一套政策”的方式强力推进。铜基新材料产业成为全省首个国家批复的区域特色高技术产业链，在建项目60个，亿元以上项目20个，投资增长100%；铜产业主营业务收入达到1060亿元，成为首个千亿元产业。半导体照明产业成为全省产业链最完整、企业数量最多、产品种类最全的产业。全省装机容量最大的中海阳光伏电站投入运行。铜拆解园等5个重点物流项目正式启动。江南文化园成为全省首批服务业集聚区。中华白姜、凤凰牡丹等特色文化旅游项目扎实起步。战略性新兴产业产值居全省第三位，增长72%，高于工业产值增幅36个百分点。
    （三）继续强化招商引资，承接产业转移有所突破。我们围绕建设承接产业转移示范市，推动各经济园区承载能力建设，累计完成基础设施投入35.4亿元。铜陵经济技术开发区成功获批国家级开发区、国家新型工业化产业示范基地。承接产业转移示范园区骨干路网、居民安置点等基础设施开工建设。各省级开发区产业承接能力进一步增强。加大招商引资力度，成立六大专业招商组，完善招商引资政策体系，深化与中央直属企业、国内知名民营企业的合作，加大对台湾地区的引资力度，开展战略性新兴产业专题招商活动30余次，台湾龙鼎等知名企业来铜落户。全年实际利用外资4亿美元，利用市外内资371.7亿元。被中国投资协会评为中国最具投资价值城市。
    （四）着力提升城市功能，城乡一体化发展步伐加快。我们强化规划引领作用，全市城乡土地利用总体规划编制完成，新一轮城市总体规划通过专家评审。长江路地下人行通道、淮河大道南段拓宽改造等工程竣工，长江二路开放通车。新增公交车辆150台，免费公共自行车交通系统正式开通。新建和改造城市污水管网100公里以上。全面完成小区整治三年计划。西湖新区在建重点项目62个，完成投资40亿元，铜博物馆、体育中心等一批重点工程进度加快。东部城区骨干路网基本拉开。南部城区政务区建成使用，铜都大道三期工程完工。铜陵长江公铁两用大桥、南京至安庆城际铁路建设进展顺利。启动了乡村公路升级改造。向天门镇供水主管网基本铺就。完成40个“千村百镇”新农村示范村、21个“村庄五化整治”示范村建设，以及农村河道清淤计划。全市耕地流转率46.5%。新增各类专业合作社88个。
    （五）深入推进改革创新，发展活力不断增强。我们全面推进社区综合管理体制改革，撤销街道一级行政机构，实现了减少层级、强化服务的目标，铜官山区成为全国首个社区管理和服务创新实验区。完善了城乡一体化改革“1+10”方案，出台了户籍制度改革12项配套政策，在全省率先发放了首批流动人口居住证，农村承包地、宅基地、房屋确权发证工作扎实开展。市对区财政管理体制改革方案出台实施。成为全国文化体制改革工作先进地区。新增国家级高新技术企业28家，高新技术产业产值增长35%。中科院皖江新兴产业技术发展中心正式组建，新设院士工作站2家、省级工程技术研究中心4家。国家铜深加工专利产业化试点基地正式挂牌，全年专利授权量740件。在全省率先启动“无线城市”建设。开展了首届市长质量奖评选活动，新增中国驰名商标1件。人才公寓一期工程进展顺利，市高技能人才公共实训基地正式挂牌，大学生创业园二期等创业载体建设加快。新增民营企业1510户、个体工商户5250户。
    （六）扎实做好节能减排工作，城市环境质量持续改善。我们强化节能预警调控和监察，实行重点用能企业能效对标，实施新亚星干熄焦等重大节能技术改造项目。强化污染物总量控制管理，推动企业实施污染减排提标改造工程，推动污水处理厂正常运行、重点排污企业达标排放。出台了建设项目环境准入管理若干规定，提高了环境准入门槛。全年淘汰落后产能企业46户，集中整治石料矿山，积极推进煤矿企业退出转型，大力推进全城区扬尘污染综合整治。全年空气质量一级天数由上年的6天增加到23天，三级天数由12天下降到2天。可吸入颗粒物日均值下降16.8%，超额完成预定指标。二氧化硫平均浓度下降5.2%。成功申报全省农村环境连片整治示范市。国家现代林业示范市建设不断深入。
    （七）精心实施民生工程，社会事业加快发展。我们在省确定的30项民生工程基础上，组织实施了48项民生工程，投入资金9.5亿元，惠及95%以上城乡居民，群众满意度超过90%。

    在全省率先实现了集中成片棚户区改造项目全面开工，棚户区改造三年计划强力推进。保障性住房建设开工率、低收入住房保障户数超额完成省政府下达的任务，保障性住房建设和管理经验在全省推广。
    在全省率先统一城乡低保标准，提高到310元/人•月。扩大失业保险基金支出范围。城镇居民养老保险全面实施，完成未参保集体企业退休人员参加基本养老保险，企业退休人员基本养老金标准提高到1309元。开展了老年人日间照料中心示范点建设，农村五保供养水平居全省前列。
    在全省率先完成36所义务教育阶段学校标准化建设任务。实施学前教育三年行动计划，新建、改建14所幼儿园，创建7所市级一类幼儿园。累计发放各类扶贫助学资金4479万元。农村留守儿童之家实现全覆盖。铜陵职业技术学院新校区建设进展顺利。
    在全省率先完成城乡卫生服务体系标准化建设。为43.9万居民建立健康档案。新型农村合作医疗人均筹资标准为全省最高。提高了医疗保险最高支付限额，建立了特大医疗费用统筹等制度，全年累计减轻职工、居民医疗费用1200余万元。
    在全省率先实现乡镇综合文化站和行政村农家书屋全覆盖，公共电子阅览室建设顺利推进，城市文化社区建设入选全国公共文化示范项目。成为全国全民健身示范试点市，成功举办2011年亚洲举重锦标赛、第二届中国安徽民俗文化节。
    在全省率先成为全国幸福家庭创建活动试点市。市地震信息中心暨防震减灾科普教育基地投入使用。妇女儿童、慈善、红十字、统计、档案、史志、民族、宗教、侨务、对台、外宣、消防、民防等各项事业都取得新进步。
    （八）努力夯实基层基础，社会大局保持和谐稳定。我们深入开展全国文明城市和国家卫生城市创建活动，组织开展了市容环境系列专项整治行动，城市管理长效机制逐步建立，城市管理的规范化、精细化水平明显提高，城乡居民的文明素质进一步增强。加强社区服务能力建设，完成7个社区工作服务用房和10个社区居民活动场所建设任务。依法治市深入开展，基层民主法制建设得到加强，法律援助工作站实现全市覆盖。坚持用群众工作统揽信访工作，实施重大事项社会风险评估制度，继续推进“四大四进四送”活动，着力化解信访积案，全市信访形势呈现“三下降一好转”的良好局面。加大治安复杂地区和突出问题专项整治，严厉打击各类刑事犯罪和经济犯罪，人民群众安全感进一步增强。安全生产形势总体平稳。食品安全监管力度加大。
    在推进经济社会发展的同时，我们切实加强政府自身建设。自觉接受人大法律监督和政协民主监督，进一步密切与工会、共青团、妇联等人民团体的联系，认真听取各方面意见和建议。认真执行人大决议，按时办结市人大代表建议171件、市政协提案311件。在部分领域实行并联审批工作模式，推行“首问办结制”、基建项目“一表清”、企业注册登记“一单联办”等制度，进入市政务服务中心的行政审批和服务事项增加到602项。行风热线、网络问政等联系群众的渠道进一步畅通，解决了一大批群众反映的实际问题。深入推进政务公开，政府信息公开成为全省先进。完善了目标管理和责任落实机制。严格贯彻落实《廉政准则》各项规定，项目审批电子监察系统开通，廉政风险防控管理全面推开，加强审计监督和效能监察，政风建设取得新成效。
    各位代表！过去一年的成绩，是在政府融资极为困难、企业经营环境不断恶化的情况下取得的，来之十分不易。这是省委、省政府和中共铜陵市委正确领导的结果，是全市人民同心同德、奋力拼搏的结果。在这里，我代表市人民政府，向全市各族人民，向广大工人、农民、知识分子、干部，向驻铜解放军指战员、武警官兵和政法干警，向各民主党派、各人民团体和社会各界人士，致以崇高的敬意！向关心、支持铜陵发展的省直各部门、兄弟市和海内外友好人士，向在我市创业的投资者、建设者，表示衷心的感谢！
    我们也清醒地认识到，我市经济社会发展还存在不少困难和问题。从外部环境看，国际经济下行风险增大，国内发展不确定因素增多，总体上仍然十分复杂严峻。从市内情况看，经济结构调整任重道远，经济运行的稳定性不高，土地总量和环境容量制约进一步加剧，影响社会和谐的一些因素依然存在。同时，少数部门和工作人员大局观念不强，服务意识淡薄，作风不够扎实，工作效率不高。对此，我们将采取有力措施加以解决，绝不辜负全市人民的期望和重托！

    二、2012年主要工作安排

    今年，是我们落实中国共产党铜陵市第九次代表大会精神的第一年。这次会议提出了建设现代产业之城、创新创业之城、精致大气之城、平安幸福之城的奋斗目标。我们一定要扎实工作，把美好蓝图一步步变为现实。
    今年，是我们提前八年全面建成小康社会的实现之年。全面建成小康社会是全市人民的共同愿望，我们要对照全面小康社会4大类21项37个指标，找出差距，明确责任，加大力度，统筹推进，切实兑现这一庄严承诺。
    今年，是本届政府的收官之年。面对更加复杂严峻的宏观环境，面对我市发展中的困难和问题，我们要坚定信心，以更加奋发有为的姿态，应对风险难题，努力化危为机，积极争先进位，力争实现圆满收官。
    今年政府工作的总体要求是：高举中国特色社会主义伟大旗帜，深入落实科学发展观，认真贯彻中央和省经济工作会议精神，按照市第九次党代会的总体部署，围绕“四个之城”建设的总体目标，以加快转变经济发展方式为主线，以推动资源型城市转型为重点，坚持稳中求进、好中求快，奋力拼抢抓承接，奋力持续抓转型，奋力争先抓创新，奋力持久抓一体，努力完成全面建成小康社会的预期目标，为推进科学发展、建设幸福铜陵，加速现代化进程奠定更加坚实的基础。
    综合考虑各种因素，今年经济社会发展主要预期目标是：全市生产总值增长13%以上；财政收入增幅高于生产总值增幅；全社会固定资产投资增长25%左右；社会消费品零售总额增长16%以上；居民消费价格指数涨幅控制在4%左右；城镇居民人均可支配收入增长15%以上，农民人均纯收入增幅超过城镇居民收入增幅；新增就业2万人，城镇登记失业率控制在3.8%以内；节能减排完成省政府下达的控制指标；人口自然增长率控制在6‰以内。
    我们将重点做好以下六个方面工作：
    （一）坚持不懈扩大项目投资，千方百计保持经济平稳较快发展。坚持投资拉动，加快传统产业技术改造，不断增强经济增长内生动力，进一步巩固和扩大经济发展良好势头。
    加大项目投资力度。抢抓内、外资项目，积极争取上级政策性投资项目；突出保续建、保重点、保民生，科学安排本级政府性投资项目。统筹安排亿元以上重点项目200个以上、10亿元以上重点项目20个以上。确保建成铜陵有色铜冶炼工艺技术升级改造、铜化大合成氨等重大项目，开工建设天奇蓝天汽车拆解等一批重点项目。深入谋划一批带动能力强的重点项目。全力抓好项目落地和竣工投产。
    继续坚持招大引强。把招商引资作为第一要事。瞄准与我市产业发展相关联的行业前20强企业、中央直属企业和国内知名民营企业，力争引进有利于提升产业竞争力、城市辐射力和集聚人气的重大项目10个以上。完善招商项目统一调度工作机制，提高招商项目履约率和开工率，全年实际利用外资增长30%，实际利用内资增长50%。市经济技术开发区基本完成全部基础设施建设，承接产业转移示范园区实现道路基本贯通、水电气配套到位，支持各县（区）开发区完善承载功能，促进各园区产业集群发展、错位竞争，在全市经济发展中切实起到引擎作用。
    做好要素保障工作。强化用地保障，把对上争取增量和对内盘活存量结合起来，严格落实投资强度规定，加大闲置土地清理力度，启动2000亩城乡建设用地增减挂钩试点，确保重大项目用地需求。加强财税金融工作。全力争取国家和省各类政策性资金。坚持有保有压、有扶有控，加强预算执行管理。清理整合各类财政专项资金，全面开展财政性资金使用情况绩效评估，充分发挥财政资金整体效益和杠杆作用。积极落实结构性减税政策，加强对小微企业的财税扶持。加强政府性基金、融资平台和债务管理。积极支持发展创业投资、风险投资。鼓励企业上市融资。抓住国家定向宽松信贷政策机遇，引导各类金融机构争取更多的信贷份额。
    继续强化企业帮扶。鼓励企业加强技术改造、产品开发和品牌培育，帮助企业做好地产品促销，促进规模以上企业做大做强，中小微企业做专做精。支持铜陵有色成为千亿元企业，铜化集团进入百亿元企业行列。密切跟踪宏观经济形势变化，强化动态监测预警，及时采取针对性的政策措施，加强煤电油运等生产要素的调度和供应保障。继续落实“百名干部进百企”等帮扶措施。
    扩大消费和进出口。加快培育文化旅游、体育休闲、家政服务、养老等消费热点，推动消费升级。加强城乡流通体系和市场基础设施建设，推进农贸市场标准化建设和管理。抓好生活必需品市场供应，实施农产品“直通车”工程。加大价格监测和监管力度，健全社会救助和保障标准与物价上涨挂钩的联动机制，绝不让低收入群众基本生活受到影响。保持房地产市场稳定健康发展。完善外贸发展促进政策，加大进出口企业孵化力度，积极申报省级服务外包基地，加快形成新的出口增长点。
    （二）围绕建设现代产业之城，加速推进产业结构转型升级。实施战略性新兴产业重大项目40个以上，力争新兴产业产值和投资增幅超过全省第一方阵的平均水平。
    全力打造世界铜都。优先发展铜基新材料，进一步延伸和完善铜基产业链，打造泛铜产业集群。促进铜陵有色25万吨高强高导铜杆、海亮二期等重点项目早日开工，加快铜拆解园等项目建设进度，促进金威铜板带等项目达产达效。推动B型保税物流中心、进口废七类转关取得实质进展，争取开源金属再生产业园列入环保部“圈区管理”，加快铜产业技术开发和产品检测、交易等产业服务平台建设，着力构筑现代铜产业体系。
    加快发展现代服务业。完善服务业发展规划，加大扶持力度，促进生产性服务业、生活性服务业加速发展，建设面向皖江城市带的商贸集散中心。加快推进示范园区物流园、皖中南物流中心等项目建设。鼓励发展第三方物流。支持科技、金融等服务业发展。实施港口码头及岸线综合整治，推行码头标准化管理，完善疏港公路。大力引进品牌商贸企业，积极推进现代服务业集聚区、特色商业街建设。继续培育社区服务业。

    加快发展文化创意产业，推动江南文化园三期、青铜帝国、国际铜文化创意产业园等重点项目建设，着力培育铜文化、民俗文化和乡村生态旅游。
    努力做强现代农业。围绕加快农业现代化进程，推进农业品牌化、农村田园化、农民市民化。完善现代农业、农村水利建设等规划，以规划引领发展，整合涉农、涉水资金和其他资源投入。推进中华白姜产业园建设，积极申报中国牡丹城，形成白姜、凤丹等特色产品系列，创立知名品牌。继续实施“菜篮子”工程，新建、改建标准化、规模化、设施化蔬菜基地3000亩。全面实施农业产业化“转型倍增计划”，加强农业科技服务，推进农业机械化，争创国家现代农业示范区。大力开展农田水利基本建设，建成高标准农田水利受益区1万亩，治理水土流失面积15平方公里。
    强力推进节能减排。实行能耗和污染物排放总量控制，强化节能减排目标管理责任制。完善能评和环评制度，严格准入条件，严格实行“一票否决”。全面推行重点领域、重点企业能耗对标评估管理，推进合同能源管理，实施20个节能改造示范工程。全面完成煤矿企业退出转型，加快淘汰落后产能。继续实施一批减排重点工程，提升污水处理厂等设施减排能力，推动有色、化工、水泥、电力等重点企业提前实施提标改造，实行清洁生产强制性审核，开展排污权交易试点。大力发展循环经济，加快循环经济工业试验园热电联产等关键节点项目建设，探索建立企业间能流、物流循环利用平台，争取成为国家循环经济示范市。
    （三）围绕建设精致大气之城，着力提升城乡建设与管理水平。坚持城乡一体，完善规划体系，增强城市功能，提升皖中南中心城市地位。建设格局大气、建设精致、管理精细、文化浓郁、宁静雅致、秀美宜居的崭新铜陵。
    提升区域交通枢纽地位。推动三大城区和各园区之间的连接，推进滨江大道南段及大通连接线、沿江公路铜陵段一级路改造，完成长江东路东村至竹园段、罗半路等工程。推动铜南宣高速复工和合巢铜高速前期工作。继续配合做好京福高铁暨铜陵长江公铁两用大桥和南京至安庆城际铁路建设。实施一批大型专业化码头项目，推进顺安河航道升级改造等项目前期工作。
    推动老城区升级改造。继续实施中心城区畅通工程，全面建成长江二路、俞家村立交桥等交通节点工程，加快北京路下穿等工程进度，建设隆门路公交换乘中心，再新增公交车辆150台。完成主城区雨污分流管网建设，全面启动城中村改造，实施社区环境提升（节能改造）工程。开工建设滨江生活岸线和滨江广场。加快推进大铜官山公园建设。完善城市管理体制机制，全面推行标准化、网格化管理。建设智慧铜陵。
    继续加快新区建设。保持西湖新区大建设态势，加快推进环西湖片区整体开发，确保19条主干路网竣工通车，体育中心建成运行。南部城区全面启动6条主干路网建设，推进祠堂湖等片区环境治理，完成九华湖旅游开发规划并力争启动实施，力争大通历史文化名镇申报成功，跨江发展有实质性进展。东部城区全线拉开路网框架，标志性建筑和配套设施建设初现形象。组织制定东部区域开发建设规划，打造东部产业新城。
    扎实推进新农村建设。完善镇（乡）、村建设规划以及公共服务设施建设规划，高标准推进中心镇和村庄综合整治工程。统筹考虑景观美化、产业发展和居民点建设，围绕建设田园化美丽乡村，启动10个以上示范村建设。全面推动5万亩土地整治示范建设项目。继续实施“千村百镇”示范、农村公路升级、危桥改造等工程，完成农村危房改造。加快实现城乡公交客运一体化运行。加快边远乡镇供水管网建设。对重点区域进行景观设计。
    大力推进生态环境治理。实施矿山生态环境恢复规划，让铜陵的破碎山体再披绿装。加大滨江区域环境综合整治和流域治理力度，加强城市饮用水水源地保护，建设应急备用水源。强化空气污染监控和治理，继续深入开展全城区扬尘污染综合整治，启动PM2.5监测。开展重金属污染治理工作，实施重金属污染治理规划。继续推进农村清洁工程。深入推进创建国家环保模范城市活动。完成东湖湿地公园和起步区规划。
    切实提高城市文化品质。深入开展社会道德和诚信教育，大力培育新时期市民精神，建立健全覆盖全社会的征信系统。开展新一轮全国文明城市创建工作，健全国家卫生城市长效管理机制。启动社区文化家园提档升级工程，力争达到全国示范水平。继续实施文化信息资源共享等一批文化惠民工程，广泛开展群众性文体活动，丰富城乡居民文化生活。高水平办好青铜文化博览会等节会，推进文化节会品牌走向世界。加快铜博物馆等文化场馆建设。
    （四）围绕建设创新创业之城，不断深化改革创新。坚持以改革的办法破解发展中的难题，切实做到人才优先，大力倡导创业光荣，加强区域创新体系建设，争创国家创业型城市。
    深入推进城乡一体化综合配套等改革。全面推行城乡一元化户籍管理制度，基本完成流动人口居住证发证工作。实现新型农村合作医疗与城镇非职工居民基本医疗保险制度并轨、新型农村社会养老保险与城镇居民社会养老保险制度并轨，形成城乡一体的促进就业（创业）政策和服务体系、社会救助“1+6”模式。启动集体用地使用权、林权、宅基地和农房抵押流转试点。稳妥有序推进事业单位分类改革。
    增强自主创新能力。加强与北大、清华等高校院所合作，推进中科院皖江新兴产业技术发展中心、产业技术创新战略联盟等创新载体建设，加快科技孵化器等创新公共服务平台建设，新建一批省、市工程技术研究中心、重点实验室，研发经费支出占地区生产总值比重达到2.5%。新增国家高新技术企业10家以上。组织实施20项自主创新重大项目，探索建立科技成果转化的激励机制。突出招才引智，制定更具吸引力的人才和创新团队引进政策。抓好人才公寓、高技能人才公共实训基地等建设。提升知识产权的创造和应用能力，全市专利申请量增长20%以上。继续推进质量强市和标准化战略，争创省级名牌5个以上。
    深入开展全民创业。继续开展千人创业扶持计划，进一步完善和落实支持创业、服务创业的政策和措施。积极推进留学生铜陵创业园、部省共建大学生创业园和农民工创业园等载体建设。大力发展非公经济和中小企业。扶持 94家省级重点调度中小企业做大做强。全年新增民营企业1500户，新增个体工商户5000户以上。
    （五）围绕建设平安幸福之城，全面推进和加强社会建设。加快基本公共服务均等化步伐，组织实施 48项民生工程，财政投入增长18%以上，进一步提高全市人民的幸福指数。
    全面实施居民收入倍增计划。实施更加积极的就业政策，多渠道开发公益性岗位，加强就业援助，确保动态消除“零就业家庭”。改进就业技能培训方式，提高城乡居民的就业技能。完善企业工资集体协商机制，落实最低工资标准，增加居民工资性收入。探索鼓励居民资产投资政策，增加居民财产性收入。提高城乡低保、医疗救助等补助标准，落实和完善强农惠农政策，增加居民转移性收入。
    切实提高住房保障水平。全面完成集中连片棚户区改造任务，实施3000户以上零星棚户区改造。新开工建设保障性住房3000套，供应1200套。完善保障性住房管理信息系统，落实保障性住房的分配、运营和管理制度。继续做好住房公积金的归集、扩面和贷款管理工作。
    提高教育和医疗服务水平。积极构筑优质、均衡的现代教育体系。实施学前教育三年行动计划，加快建立覆盖城乡、布局合理的学前教育公共服务体系。进一步优化城乡义务教育布局，完善校长和教师交流制度，推动中小学数字化校园建设。着力解决校车安全问题。促进普通高中优质化、多元化、特色化发展。整合职教资源，加快建设皖中南职教基地。打造区域医疗卫生服务中心。强化基层医疗卫生机构内涵建设，完成新组建社区卫生服务机构建设，完善城乡居民健康档案等服务机制，提升公共医疗卫生保障水平。抓好重大传染病防治和地方病防控。
    统筹发展各项社会事业。扎实推进全国全民健身示范市创建，办好安徽省第三届体育大会。全面推进幸福家庭创建工作，努力实现流动人口与城市居民同城同待遇。进一步完善公共养老服务体系，推进养老服务社会化，力争走在全国前列。积极争创国家残疾人无障碍示范市和全省司法行政工作示范市。继续加强外事、侨务、民族、宗教、对台、气象、防震减灾、档案、史志、慈善、红十字会等工作。充分发挥工会、共青团、妇联等人民团体作用。
    巩固扩大社区综合管理体制改革成果。完善社区居民自治制度，引导社区居民参与社区管理。实行社区管理服务准入制，强化社区共驻共建工作。加强社区服务用房和居民活动场所建设，满足社区服务和居民活动需要。改革社区公共服务模式，开展政府购买社会组织服务试点，积极培育发展各类社会组织。努力打造一支专业化的社区专职工作者队伍。加大农村社区建设推进力度，争创全国农村社区建设实验全覆盖示范单位。
    切实维护社会和谐稳定。做好新形势下的群众工作，进一步完善群众权益保障、诉求表达、利益协调和矛盾纠纷排查化解等机制，切实维护群众的合法权益。继续推进“四大四进四送”活动，健全重大事项风险评估制度，强化信访源头治理，坚决防止和妥善处置群体性事件。高度重视网络舆情，加强互动和疏导，营造健康平和的社会心态。完善社会治安综合防控体系，加大打黑除恶力度，维护社会平安和谐。毫不松懈地抓好安全生产，全面推行企业安全生产标准化管理，坚决防止重特大事故发生。强化食品、药品全程安全监管，提高保障公共安全和应对突发事件的能力，确保人民群众生命财产安全。
    （六）加强政府自身建设，切实提升政府执行力。全面完成今年的各项目标任务，必须加强行政能力建设，努力建设依法高效、负责务实、人民满意的政府。
    坚持不断学习，增强发展本领。扎实推进学习型政府建设，政府工作人员特别是领导干部要认真钻研业务，努力成为行家里手，着力提高科学谋划水平。加强对国际国内形势的跟踪和研判，超前谋划并做好与上级政策的衔接和落实，不断提高解决实际问题的能力。
    严格依法行政，规范政府行为。自觉接受人大法律监督、政协民主监督、人民群众和新闻媒体的监督，认真落实人大决议，办理好人大议案、代表建议和政协提案。深入推进规范行政执法示范市创建工作，促进公正执法、文明执法。完善重大事项决策咨询、公示、听证等制度，加强对重大政策实施情况的跟踪评估和问效，不断提高决策科学化和民主化水平。
    推进管理创新，优化政府服务。组织开展对行政审批事项再次清理，完善首问办结制和并联审批模式。全面实施行政审批电子监察。加大事关群众利益、百姓关注的政府信息公开力度，更好发挥行风热线、市民论坛等在联系群众、服务百姓方面的平台作用。加强电子政务和政务服务中心建设，努力营造办事最快、成本最低、服务最优的政务环境。
    深化作风建设，提升行政效能。牢固树立公仆意识和正确的政绩观，多办顺民意、解民忧、增民利的实事，多做谋长远、打基础、利全局的工作。坚持实干兴铜，健全工作落实机制，把任务一项一项抓实，把事情一件一件办好。健全目标管理责任体系，建立对县区（园区）分类考核机制，加大政务督查和行政问责力度。深入推进机关效能建设，大力精简会议和文件，进一步形成干事业、抓落实、促发展的良好氛围。
    加强反腐倡廉，建设廉洁政府。认真落实党风廉政建设责任制和领导干部廉洁自律各项规定，扎实推进廉政风险防控管理工作，不断强化惩治和预防腐败体系建设。坚持用制度管权、管事、管人，加强对财政资金、重大投资项目和民生事项的审计，深入开展工程建设领域突出问题专项治理，严肃查处违法违纪行为，坚决纠正损害群众利益的不正之风。深入推进节约型机关建设，积极探索职务消费改革，严格控制“三公”消费，努力降低行政运行成本，始终保持勤政为民、务实清廉。
    各位代表！铜陵正处在转型跨越的关键时期，铜都大地春雷激荡，铜都儿女豪情满怀。让我们在中共铜陵市委的坚强领导下，紧紧依靠和团结全市人民，同心同德，奋发进取，扎实工作，为顺利完成全面建成小康社会目标，为建设现代产业之城、创新创业之城、精致大气之城、平安幸福之城而努力奋斗！
